# Comb Model in Periodic Potential

**DOI:** 10.3390/e28020165

**Published:** 2026-01-31

**Authors:** Alexander Iomin, Alexander Milovanov, Trifce Sandev

**Affiliations:** 1 Solid State Institute, Technion—Israel Institute of Technology, Haifa 32000, Israel; iomin@g.technion.ac.il; 2 Max Planck Institute for the Physics of Complex Systems, 01187 Dresden, Germany; alexander.milovanov@enea.it; 3 ENEA National Laboratory, Centro Ricerche Frascati, I-00044 Frascati, Rome, Italy; 4 Research Center for Computer Science and Information Technologies, Macedonian Academy of Sciences and Arts, Bul. Krste Misirkov 2, 1000 Skopje, Macedonia; 5 Institute of Physics, Faculty of Natural Sciences and Mathematics, Ss. Cyril and Methodius University, Arhimedova 3, 1000 Skopje, Macedonia; 6 Department of Physics, Korea University, Seoul 02841, Republic of Korea

**Keywords:** subdiffusion, periodic potential, non-equilibrium stationary state, Mathieu function, Fox H-functions

## Abstract

A comb model with periodic potential in side branches is introduced. A comb model is a model of geometrically constrained diffusion, such that the diffusion process along the comb’s main axis (backbone) is coupled to the diffusion process in fingers, the side branches of the comb. Here, we consider a generalized version of this complex process by enabling a periodic potential function in the fingers. We aim to understand how the potential function added affects the asymptotic transport scalings in the backbone. A set of exact results pertaining to the generalized model is obtained. It is shown that the relaxation process in fingers leads directly to the occurrence of a non-equilibrium stationary state (NESS) in comb geometry, provided that the total energy is zero. Also, it is shown that the spatial distribution of the probability density in proximity to NESS is given by the Mathieu distribution with zero energy. The latter distribution is found to be the direct result of relaxation towards stationarity of the Mathieu eigenspectrum. It is suggested that the generalized model can characterize anisotropic particle dispersion in beta-plane atmospheric (alternatively, electrostatic drift-wave plasma) turbulence and the subsequent formation of layered structures, zonal flows, and staircases. In this regard, the inherent interconnection between combs and staircases is discussed in some detail.

## 1. Introduction

**Figure 1:**
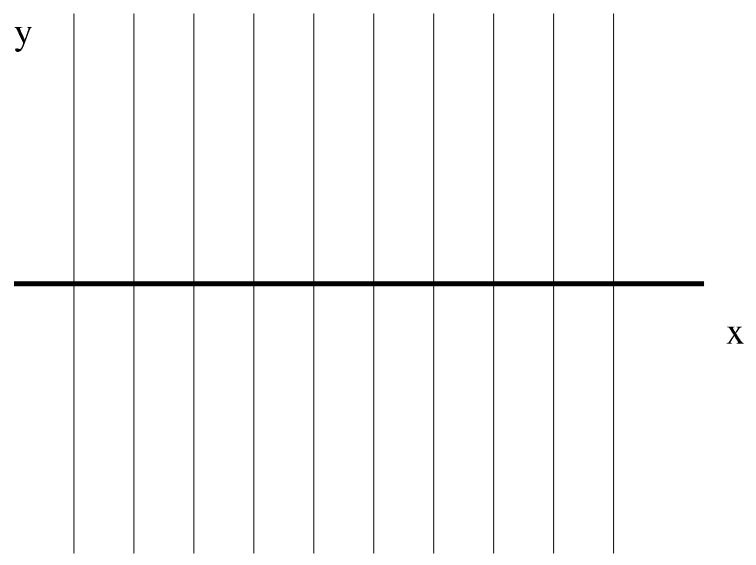
The comb structure. The comb’s fingers, or teeth (*y* axis), are continuously and uniformly distributed along the backbone (*x* axis).

A comb model, being a specific realization of random loopless structures, is a model of geometrically constrained diffusion. A typical comb, shown in Figure 1, consists of a central backbone along the *x* axis and infinite side branches (teeth or fingers of the comb) in the *y* direction. Because combs are loopless objects (similarly to Bethe lattices), they have long been considered as an appealing simplification of percolation clusters [1,2,3]. A remarkable feature about combs is that they capture much of the actually observed signatures of anomalous transport in disordered systems, with side branches being the sources for memory and dynamical traps. In this context, the comb model could be regarded as a geometric representation of the continuous-time random walk [3]. The various aspects of anomalous diffusion on combs have been discussed in [4,5,6,7,8,9,10,11,12,13]. The possible physics applications are reviewed in [14,15,16,17]. A common feature of all these applications is the understanding that the classic random-walk process in comb geometry leads to asymptotic subdiffusion along the backbone, with the mean-squared displacement (MSD) growing with time as $t^{1/2}$. This subdiffusive scaling law has been found in a variety of experimental situations and mathematical models, with theoretical underpinnings pertaining to the percolation problem, the first passage time density problem, trapping-induced diffusion-controlled reactions, interactions through soft pairwise potentials considered within the linearized Dean–Kawasaki framework, and Lévy flights in optics [18,19,20,21,22,23,24]. Among other applications, the finite velocity transport [20,25,26], quenched and annealed disorder mechanisms [27] and non-Markovian quantum phenomena [28] should be admitted as well. Part of the challenge is concerned with biomedical aspects, such as fractional oncology [29,30] and fractional neurology [31,32,33]. Other issues include stochastic resetting with non-equilibrium stationary states realization [34], the various generalizations of the Ornstein–Uhlenbeck process [35,36], and random walks on uniform and non-uniform combs and brushes [12,13,37,38]. Another area of attraction is the one related to layering phenomena in drift–wave plasma (alternatively, beta–plane atmospheric) turbulence [39,40,41,42,43,44,45,46]; see also Ref. [47] for review. The main idea here is that poloidal and radial diffusion in toroidal magnetic confinement systems such as tokamaks and stellarators may couple together to produce long-lived regular patterns of highly concentrated jet zonal flows, the so-called plasma staircase [39,40,41,42,43,44,45], with the coupling agent being identified as turbulence spreading [46].

Here, we consider a simplified version of this complex process and generalize the comb model due to Arkhincheev and Baskin [48] by augmenting it with a periodic potential in fingers. We aim to understand how the potential function added affects the asymptotic transport scalings in the backbone. Subsequently, we show that the relaxation process in fingers leads to a possibility of a non-equilibrium stationary state (NESS) in a comb geometry. The latter distribution is found to be the direct result of relaxation towards stationarity of the Mathieu eigenspectrum.

A mathematically elegant way of the comb model has been suggested in Ref. [48] in the form of the two-dimensional Fokker–Planck equation, which is discussed in the next section. Our aim is to consider the comb model in a periodic potential V(y+a)=V(y). For our purpose, we first consider some variation of the comb model, a so-called half-plane comb model, when V(y)=0. To that end, in Section 2, we consider some new results in the framework of the half-plain comb model to shed light on the geometrical description of subdiffusion. In Section 3, the periodic potential is introduced along the *y* axis. In this case, the length of the fingers can be restricted by the period of the potential, |y|≤a. Usually, this additional topological restriction leads to normal diffusion along the backbone [49]. However, in the presence of the periodic potential, subdiffusion does take place along the backbone. This situation leads to a new possibility of controlling the backbone transport in the comb models in external fields.

## 2. Half-Plane Comb Model

Let P(x,y,t) be the probability density function (PDF) of a particle/tracer to be in position with coordinates (x,y) at time *t*. Then the half-plane comb model reads

(1) $$\frac{\partial }{\partial t}P\left(x,y,t\right)=\delta \left(y\right)\frac{\partial^{2}}{\partial x^{2}}P\left(x,y,t\right)+d\frac{\partial^{2}}{\partial y^{2}}P\left(x,y,t\right).$$

Here, $x\in R_{+}\left(0,\infty \right)$ corresponds to the backbone, while y∈R(−∞,∞) are side branches (teeth or fingers) continuously distributed along the backbone. All variables and parameters are taken in dimensionless form, so *d* is an effective diffusion coefficient in the *y* direction, while the diffusion coefficient in the *x* direction is the Dirac delta function δ(y). In this case, diffusion in the *x* direction is possible at y=0 only. The boundary conditions at infinity are taken to be zero, namely $P\left(x=+\infty ,y,t\right)=\partial_{x}P\left(x=+\infty ,y,t\right)=0$ and $P\left(x,y=\pm \infty ,t\right)=\partial_{y}P\left(x,y=\pm \infty \right)=0$. There are various scenarios of the initial and boundary conditions at x=0, which will be specified in what follows in the text. The boundary conditions at x=0 will be defined in the subsequent scenarios.

### 2.1. Half-Plane Comb Model I

Let us first consider the initial condition at t=0, defined as $P_{0}\left(x,y\right)=C\left(x\right)\delta \left(y\right)$, where C(x)=0,x>0. The boundary condition on the backbone at x=0 is given by $P\left(x=0,y=0,t\right)=C_{0}$, where $C_{0}=\mathrm{const}$ for t>0; that is, the boundary condition is imposed at the initial point of the backbone. In this situation, it is better to discuss the density function (DF) of transporting particles. To some extent, this scenario corresponds to a variation of a standard situation; see, e.g., [50]. Performing the Laplace transform with respect to time, $\hat{P}\left(x,y,s\right)=L\left[P(x,y,t)\right]\left(s\right)$, we have Equation (1) as follows

(2) $$s\hat{P}\left(x,y,s\right)=\delta \left(y\right)\frac{\partial^{2}}{\partial x^{2}}\hat{P}\left(x,y,s\right)+d\frac{\partial^{2}}{\partial y^{2}}\hat{P}\left(x,y,s\right).$$

At the boundary x=0, we have

(3) $$\hat{P}\left(x=0,y=0,s\right)=\int_{0}^{\infty }e^{-st}P\left(x=0,y=0,t\right)=\frac{C_{0}}{s}.$$

In Laplace space, the transport along the *x* and *y* directions is independent. Then we look for the solution to Equation (2) in the form of the following ansatz

(4) $$\hat{P}\left(x,y,s\right)=e^{-\left|y\right|\sqrt{s/d}}f\left(x,s\right)\equiv e^{-\left|y\right|\sqrt{s/d}}\hat{P}\left(x,y=0,s\right),$$

where $\hat{P}\left(x,y=0,s\right)$ is the Laplace image of the backbone density.

Let us obtain the equation for the marginal density of the *x* transport. It reads as follows

(5) $$P_{1}\left(x,t\right)=\int_{-\infty }^{\infty }P\left(x,y,t\right)dy.$$

Performing integration with respect to *y* in Equations (2) and (4), and taking into account the boundary conditions at infinity and Equations (2) and (3), we obtain the following equations for the marginal DF

(6) $$\begin{array}{cc} s{\hat{P}}_{1}\left(x,s\right) & =\frac{\partial^{2}}{\partial x^{2}}{\hat{P}}_{1}\left(x,y=0,s\right), \end{array}$$

(7) $$\begin{array}{cc} f(x=0,s) & =\hat{P}\left(x=0,y=0,s\right)=\frac{C_{0}}{s}. \end{array}$$

Equation (7) also means that $C_{0}=1$. Then Equation (4) yields

(8) $${\hat{P}}_{1}\left(x,s\right)=\hat{P}\left(x,y=0,s\right)\int_{-\infty }^{\infty }e^{-\left|y\right|\sqrt{s/d}}dy=2{\left[d/s\right]}^{1/2}f\left(x,s\right).$$

Substituting the relation (8) in Equation (6) and taking into account the boundary condition (7) for the backbone DF, we obtain it in Laplace space as follows:

(9) $$f\left(x,s\right)=\frac{C_{0}}{s}\exp\left[-{\left(4sd\right)}^{1/4}x\right].$$

Therefore, the marginal DF in Laplace space is

(10) $${\hat{P}}_{1}\left(x,s\right)=2{\left[d/s\right]}^{\frac{1}{2}}f\left(x,s\right)=\frac{2C_{0}d^{1/2}}{s^{3/2}}\exp\left[-{\left(4sd\right)}^{1/4}x\right].$$

To estimate the MSD according to the backbone DF, one needs to normalize the latter, since it is the distribution of the non-conserved number of particles, which increases with time due to the boundary condition at x=0. Therefore, the number of particles in Laplace space N(s) is the normalization constant, which reads

(11) $$N\left(s\right)=\int_{0}^{\infty }f\left(x,s\right)dx=\frac{C_{0}}{{\left(4d\right)}^{1/4}}s^{-5/4}.$$

Consequently, the normalization reads

$$N\left(t\right)=L^{-1}\left[N(s)\right]=\frac{C_{0}t^{1/4}}{{\left(4d\right)}^{1/4}\Gamma \left(5/4\right)}.$$

Therefore, the MSD along the backbone is

(12) $$\langle x^{2}\left(t\right)\rangle =\frac{1}{N(t)}L^{-1}\left[\int_{0}^{\infty }x^{2}f\left(x,s\right)dx\right]=\frac{2\Gamma \left(5/4\right){\left(t/d\right)}^{1/2}}{\Gamma (7/4)}.$$

Eventually, the two-dimensional DF of the half-plane comb model (3) is

(13) $$P\left(x,y,t\right)=C_{0}\;L^{-1}\left[e^{-{|y|\left(s/d\right)}^{1/2}}s^{-1}\;e^{-{\left(4sd\right)}^{1/4}x}\right].$$

This integration leads to the convolution of the Gaussian distribution [50] and the Fox *H*-function [51]; see Appendix B.

An additional correction of the diffusion coefficient for the MSD (12) can also be performed due to the two-dimensional geometry. The number of transporting particles according to the DF (13) is

(14) $$\begin{array}{cc} N_{2D}\left(t\right) & =L^{-1}\left[\;\int \int \;e^{-|y|(s/d^{1/2})}\frac{1}{s}e^{-{\left(4sd\right)}^{1/4}x}dxdy\right]\\ \; & =L^{-1}\left[{\frac{C_{0}}{d}}^{1/4}\sqrt{2}\;s^{7/4}\right]=\frac{C_{0}d^{1/4}t^{3/4}}{{\left(4d\right)}^{1/4}\Gamma \left(7/4\right)}. \end{array}$$

Then the MSD, according to the 2D PDF, is

(15) $$\langle x^{2}\left(t\right)\rangle =\frac{1}{N_{2D}\left(t\right)}L^{-1}\left[\int_{0}^{\infty }x^{2}P_{1}\left(x,s\right)dx\right]=\frac{2\Gamma \left(7/4\right){\left(t/d\right)}^{1/2}}{\Gamma (9/4)}.$$

### 2.2. Half-Plane Comb Model II

Let us now consider the half-plane comb model with the non-zero initial condition $P\left(x,y,t=0\right)=P_{0}\left(x,y\right)=F\left(x\right)\delta \left(y\right)$, and zero boundary condition at x=0, namely P(x=0,y,t)=0. In this case, the strategy of the solution is to determine the Green function G(x,y,t) for the standard two-dimensional comb model, with an odd initial condition ϕ(x,y) imposed on the entire *x* axis; that is, ϕ(x=0,y)=0 and, correspondingly, F(−x)=−F(x). Then, the solution for the PDF of the half-plane comb model is the convolution

(16) $$\begin{array}{cc} P(x,y,t) & =\overset{\infty }{\underset{-\infty }{\;\int \int \;}}G\left(x-x^{\prime },y-y^{\prime },t\right)\phi \left(x^{\prime },y^{\prime }\right)dx^{\prime }dy^{\prime }, \end{array}$$

(17) $$\begin{array}{cc} \phi (x,y) & =\left\{\begin{array}{cc} F(x)\delta (y)\;\; & \mathrm{for}\;\;x>0,\\ 0\;\; & \mathrm{for}\;\;x=0,\\ F(x)\delta (y)\;\; & \mathrm{for}\;\;x<0. \end{array}\right. \end{array}$$

Therefore, the backbone transport can be described by the marginal PDF $P_{1}\left(x,t\right)=\int P\left(x,y,t\right)dy$ and, correspondingly, by the Green function (the marginal transition probability) $G_{1}\left(x,t\right)=\int G\left(x,y,t\right)dy$ with the initial condition $\phi_{1}\left(x\right)=\int \phi \left(x,y\right)dy$, according to Equations (16) and (17). It is worth noting that, in this boundary value problem, the number of particles is conserved. Then, the backbone solution (16) for the half-plane comb model can be presented by the chain of simple transformations,

(18) $$\begin{array}{cc} P_{1}\left(x,t\right) & =\int_{-\infty }^{\infty }G_{1}\left(x-x^{\prime },t\right)\phi_{1}\left(x^{\prime }\right)dx^{\prime }\\ \; & =\int_{0}^{\infty }G_{1}\left(x-x^{\prime },t\right)F\left(x^{\prime }\right)dx^{\prime }+\int_{-\infty }^{0}G_{1}\left(x-x^{\prime },t\right)F\left(x^{\prime }\right)dx^{\prime }\\ \; & =\int_{0}^{\infty }\left[G_{1}\left(x-x^{\prime },t\right)-G_{1}\left(x+x^{\prime },t\right)\right]F\left(x^{\prime }\right)dx^{\prime }, \end{array}$$

where F(−x)(−dx)=F(x)dx. Then the Fourier transform with respect to *x* yields

(19) $$\begin{array}{cc} {\tilde{P}}_{1}\left(k,t\right) & =\int_{-\infty }^{\infty }e^{-ikx}P_{1}\left(x,t\right)dx={\tilde{G}}_{1}\left(k,t\right)\int_{0}^{\infty }F\left(x^{\prime }\right)\left[e^{-ikx^{\prime }}-e^{+ikx^{\prime }}\right]dx^{\prime }\\ \; & ={\tilde{G}}_{1}\left(k,t\right)\left[\int_{0}^{\infty }e^{-ikx^{\prime }}F\left(x^{\prime }\right)dx^{\prime }-\int_{0}^{-\infty }e^{-ikx}F\left(-x^{\prime }\right)\left(-dx^{\prime }\right)\right]\\ \; & ={\tilde{G}}_{1}\left(k,t\right)\int_{-\infty }^{\infty }F\left(x^{\prime }\right)e^{-ikx^{\prime }}dx^{\prime }={\tilde{G}}_{1}\left(k,t\right)\tilde{F}\left(k\right). \end{array}$$

### 2.3. Green Function

Let us first solve the equation for the comb Green function G=G(x,y,t) with the initial condition $G_{0}=\delta \left(x-x_{0}\right)\delta \left(y-y_{0}\right)$. Then, the standard comb equation for the Green function reads

(20) $$\frac{\partial }{\partial t}G\left(x,y,t\right)=\delta \left(y\right)\frac{\partial^{2}}{\partial x^{2}}G\left(x,y,t\right)+d\frac{\partial^{2}}{\partial y^{2}}G\left(x,y,t\right).$$

The zero boundary conditions are at infinity.

Performing the Laplace transform and looking for the solution in the form of an ansatz (4), we have

(21) $$\hat{G}\left(x,y,s\right)=e^{-{|y|\left(s/d\right)}^{1/2}}g\left(x,s\right),$$

where $g\left(x,s\right)=\hat{G}\left(x,y=0,s\right)$. Integrating Equations (20) and (21) with respect to *y*, where $G_{1}\left(x,t\right)=\int G\left(x,y,t\right)dy$, we have in Laplace space

(22) $$s{\hat{G}}_{1}\left(x,s\right)-\delta \left(x-x_{0}\right)=\frac{\partial^{2}}{\partial x^{2}}g\left(x,s\right)$$

and

(23) $${\hat{G}}_{1}\left(x,s\right)=2{\left(d/s\right)}^{1/2}\;g\left(x,s\right).$$

Then, replacing g(x,s) in Equation (22) by means of Equation (23) and performing the Fourier transform, we obtain the *x*-Green function in Fourier–Laplace space as follows:

(24) $${\hat{\tilde{G}}}_{1}\left(k,s\right)=\frac{s^{-1/2}}{s^{1/2}+k^{2}/\left[2\sqrt{d}\right]},$$

where we set $x_{0}=0$. Then, the Fourier image of the Green function is expressed in the form of the Mittag–Leffler function [52],

(25) $$G_{1}\left(k,t\right)=E_{1/2}\left(-k^{2}t^{1/2}/\left[2\sqrt{d}\right]\right)=\sum_{n=0}^{\infty }\frac{{\left(-k^{2}t^{1/2}/\left[2\sqrt{d}\right]\right)}^{n}}{\Gamma (1+n/2)}.$$

### 2.4. Half-Plane Comb Model II: MSD

Taking into account Equation (19), we obtain the Fourier–Laplace image of the marginal PDF of the half-plane comb model as follows:

(26) $${\hat{\tilde{P}}}_{1}\left(k,s\right)={\hat{\tilde{G}}}_{1}\left(k,s\right)\tilde{F}\left(k\right)=\frac{s^{-1/2}\tilde{F}\left(k\right)}{s^{1/2}+k^{2}/\left[2\sqrt{d}\right]}.$$

The inverse Fourier–Laplace transform yields

(27) $$\begin{array}{c} P_{1}\left(x,t\right)=\int_{-\infty }^{\infty }\frac{1}{2|x-\xi |}H_{1,1}^{1,0}\left[\frac{\left|x-\xi \right|}{{\left(t^{1/2}/\left[2\sqrt{d}\right]\right)}^{1/2}}\left|\begin{array}{c} \left(1,1/4\right)\\ \left(1,1\right) \end{array}\right.\right]F\left(\xi \right)\;d\xi . \end{array}$$

Here, the Fox *H*-function $H_{1,1}^{1,0}\left(z\right)$ (A4) is obtained by means of the Mellin–Barnes integral; see Appendices Appendix A and Appendix B.

As admitted above, in the second scenario of the half-plane comb model, the number of particles is conserved. The latter is determined by the initial condition $N=\tilde{F}\left(0\right)$. From Equation (26) we have

$$N=L^{-1}{\hat{\tilde{P}}}_{1}\left(k=0,s\right)=\theta \left(t\right)\tilde{F}\left(k=0\right)=\tilde{F}\left(0\right),$$

which is the normalization constant for the PDF, where θ(t) is the Heaviside theta function.

## 3. Side-Branched Periodic Potential

As shown above, the consideration of the half-plane comb model eventually reduces to the standard analysis of the two-dimensional comb model, where the boundary conditions on the backbone at x=0 are unnecessary and can be discarded. In this case, x∈R(−∞,∞) corresponds to the backbone. Let P(x,y,t) be the PDF of a particle/tracer being at the position with coordinates (x,y) at time *t*. Then, the comb model in a periodic potential V(y+a)=V(y) reads

(28) $$\frac{\partial }{\partial t}P\left(x,y,t\right)=\delta \left(y/a\right)D_{1}\frac{\partial^{2}}{\partial x^{2}}P\left(x,y,t\right)+D_{2}\frac{\partial^{2}}{\partial y^{2}}P\left(x,y,t\right)-\frac{\partial }{\partial y}\left[V^{\prime }\left(y\right)P\left(x,y,t\right)\right],$$

where $D_{1}\delta \left(y/a\right)$ and $D_{2}$ are diffusion coefficients in the *x* and *y* directions, respectively. The periodic potential is defined as $V\left(y\right)=V_{0}\cos\left(\frac{2\pi }{a}y\right)$, the prime means derivative with respect to *y*, and $V_{0}$ is the amplitude of the periodic variation. Here, *a* is the period of the potential, which describes the effective length of the side branches.

All variables and parameters can be taken in the same dimensionless form as in Equation (1) by introducing the scaling parameters $T=a^{2}/D_{1}$ for time, with t/T→t and L=a for the spatial coordinates, with x/L→x, and $y/L\to y/\sqrt{d}$, and $d=D_{2}/D_{1}$. Note that $D_{1}$ has the dimension of the diffusion coefficient as well. The amplitude of the periodic potential can also be considered in dimensionless form by scaling $V_{0}/D_{1}\to V_{0}$. The latter scaling reflects the fact that the dimensionless $V_{0}\ll 1$. After rescaling, the periodic potential reads $V\left(y\right)=V_{0}\cos\left(2\pi y\right)$. Eventually, the dimensionless form of Equation (28) reads

(29) $$\frac{\partial }{\partial t}P\left(x,y,t\right)=\delta \left(y\right)\frac{\partial^{2}}{\partial x^{2}}P\left(x,y,t\right)+d\frac{\partial^{2}}{\partial y^{2}}P\left(x,y,t\right)-\frac{\partial }{\partial y}\left[V^{\prime }\left(y\right)P\left(x,y,t\right)\right].$$

The boundary conditions at infinity are taken to be zero, namely $P\left(x=\pm \infty ,y,t\right)=\partial_{x}P\left(x=\pm \infty ,y,t\right)=0$ and $P\left(x,y=\pm \infty ,t\right)=\partial_{y}P\left(x,y=\pm \infty \right)=0$. Note also that the side-branched boundary conditions in the presence of the periodic potential will be redefined as necessary; namely, these boundaries can be considered at half of the period of the periodic potential. The initial condition now is $P\left(x,y,t=0\right)=P_{0}\left(x,y\right)=\delta \left(x\right)\delta \left(y\right)$.

Introducing a new PDF [53,54]

(30) $$P\left(x,y,t\right)=e^{-V(y)/[2d]}\bar{P}\left(x,y,t\right)$$

and substituting it in Equation (29), we obtain the following equation:

(31) $$\frac{\partial }{\partial t}\bar{P}\left(x,y,t\right)=\delta \left(y\right)\frac{\partial^{2}}{\partial x^{2}}\bar{P}\left(x,y,t\right)+d\frac{\partial^{2}}{\partial y^{2}}\bar{P}\left(x,y,t\right)-V_{J}\left(y\right)\bar{P}\left(x,y,t\right),$$

where $V_{J}\left(y\right)={\left[V^{\prime }\left(y\right)\right]}^{2}/4d-3V^{\prime \prime }\left(y\right)/2$. In particular, if $V\left(y\right)=V_{0}\cos\left(2\pi y\right)$, then

(32) $$V_{J}=\frac{\pi^{2}V_{0}^{2}}{d}\sin^{2}\left(2\pi y\right)+6\pi^{2}V_{0}\cos\left(2\pi y\right).$$

Following the standard procedure [16,48], we perform the Laplace transform of Equation (31) with respect to time and look for the Laplace image in the form of the ansatz

(33) $$\hat{\bar{P}}\left(x,y,s\right)=g\left(y,s\right)f\left(x,s\right)=e^{-\left|y\right|\sqrt{s/d}}\chi \left(y,s\right)f\left(x,s\right).$$

Note that this ansatz differs from the standard one defined in Equations (4) and (21) by an additional side-branched function χ(y,s). Substituting Equation (33) in the Laplace transform of Equation (31), we obtain the following equation:

(34) $$\begin{array}{cc} 0 & =\delta \left(x\right)\delta \left(y\right)+\delta \left(y\right)e^{-\left|y\right|\sqrt{s/d}}\chi \left(y,s\right)\frac{\partial^{2}f\left(x,s\right)}{\partial x^{2}}-2\sqrt{sd}\delta \left(y\right)e^{-\left|y\right|\sqrt{s/d}}\chi \left(y,s\right)f\left(x,s\right)\\ \; & -2\sqrt{sd}\;\mathrm{sgn}\left(y\right)\left[\frac{\partial \chi (y,s)}{\partial y}\right]e^{-\left|y\right|\sqrt{s/d}}f\left(x,s\right)+d\left[\frac{\partial^{2}\chi \left(y,s\right)}{\partial y^{2}}\right]e^{-\left|y\right|\sqrt{s/d}}f\left(x,s\right)\\ \; & -V_{J}\left(y\right)e^{-\left|y\right|\sqrt{s/d}}\chi \left(y,s\right)f\left(x,s\right), \end{array}$$

where sgn(y) is the sign function of *y*. This equation can be divided into two equations by separating terms with the Dirac delta function δ(y) that corresponds to the backbone transport at y=0 from the terms valid for y∈R(−∞,∞) that correspond to the side-branched transport ∀x. Thus, we have

(35) $$\frac{\partial^{2}f\left(x,s\right)}{\partial x^{2}}-2\sqrt{sd}f\left(x,s\right)+\delta \left(x\right)/\chi \left(0\right)=0,$$

which is the backbone equation, where χ(0)≡χ(y=0,s). The second equation follows immediately from Equation (34) for y≠0. It determines an additional χ(y,s) function, which appears in the analysis due to the periodic potential $V_{J}\left(y\right)$ and for f(x,s)≠0 it reads

(36) $$d\frac{\partial^{2}\chi \left(y,s\right)}{\partial y^{2}}-2\sqrt{sd}\;\mathrm{sgn}\left(y\right)\;\frac{\partial \chi (y,s)}{\partial y}-V_{J}\left(y\right)\chi \left(y,s\right)=0.$$

First, we consider the backbone transport.

### 3.1. Backbone Transport

Performing the Fourier transform of the backbone Equation (35), we have

(37) $$\begin{array}{c} -k^{2}\tilde{f}\left(k,s\right)-2\sqrt{sd}\tilde{f}\left(k,s\right)+1/\chi \left(0\right)=0 \end{array}$$

that yields

(38) $$\begin{array}{c} \tilde{f}\left(k,s\right)=\frac{1/\chi (0)}{k^{2}+2\sqrt{sd}}. \end{array}$$

Performing the Fourier inversion, we obtain

(39) $$\begin{array}{c} f\left(x,s\right)=\frac{e^{-{|x|\left(4sd\right)}^{1/4}}}{2\sqrt{2\sqrt{sd}}\chi \left(0\right)}. \end{array}$$

Substituting solution (39) in Equation (35), one verifies the correctness of the analysis.

### 3.2. Side-Branched Transport: Mathieu Equation

The side-branched Equation (36) is symmetrical with respect to inversion y→−y. Therefore, for y>0, it reads in the form of the ordinary differential equation as follows:

(40) $$\chi^{\prime \prime }\left(y\right)-2\sqrt{s/d}\;\chi^{\prime }\left(y\right)-\frac{1}{d}V_{J}\left(y\right)\chi \left(y\right)=0.$$

To simplify the consideration without restriction of generality, we take into account that in the periodic potential $V_{J}\left(y\right)$, the parameter $V_{0}/d\ll 1$ is small. Then, neglecting the term with $V_{0}^{2}$ in Equation (32), we obtain the following:

(41) $$\chi^{\prime \prime }\left(y\right)-2\sqrt{s/d}\;\chi^{\prime }\left(y\right)-\pi^{2}\gamma_{1}\cos\left(2\pi y\right)\chi \left(y\right)=0,$$

where $\gamma_{1}=6V_{0}/d\ll 1$. Note that this simplification of $V_{J}$ does not change its period. To arrive at the Mathieu equation, we make the substitution $\chi \left(y\right)=e^{\sqrt{s/d}y}u\left(y\right)$ and then define z=πy. That yields

(42) $$u^{\prime \prime }\left(z\right)+\left[\left(-s/\pi^{2}d\right)-\gamma_{1}\cos\left(2z\right)\right]u\left(z\right)=0.$$

Note also, considering this equation symmetrically, the second derivative of the term $\frac{d^{2}}{dy^{2}}\left[e^{\left|y\right|\sqrt{s/d}}\right]$ does not contain the delta-function, since δ(y)=0 for y≠0.

Following the standard definition [55,56], we arrive at the Mathieu equation:

(43) $$\frac{d^{2}u\left(z\right)}{dz^{2}}+\left[\alpha -2q\cos(2z)\right]u\left(z\right)=0,$$

where $\alpha =-s/\pi^{2}d$ and $2q=\gamma_{1}$. Floquet theorem [55,56], ensures the solution

(44) $$u\left(z\right)=e^{i\nu z}w\left(z\right),\;w\left(z+\pi \right)=w\left(z\right)$$

with the property $u\left(z+\pi \right)=e^{i\nu \pi }u\left(z\right)$, where ν is the quasi-energy (also known as the characteristic exponent). It relates to the eigenvalues of the Mathieu Equation (43) as follows [55,56]: $\alpha =\lambda_{\nu }\left(q\right)=\nu^{2}+q^{2}/2\left(\nu^{2}-1\right)+o\left(q^{2}\right)$. This situation is unique when the Mathieu spectrum $\lambda_{\nu }=\alpha \propto s$ is proportional to the inverse time and tends to zero with time as s→0. Considering the large-time dynamics when $s/\pi^{2}d\ll 1$, we find that, for α,q≪1, the only reasonable solution is the Mathieu function ${\mathrm{ce}}_{0}\left(z,q\right)$, which is positive for z∈R(−∞,∞) and has the spectrum $\alpha =-q^{2}/2\to -0$ [55,56,57]. However, without restriction of the transport properties, we restrict the dynamics by boundaries z∈[−πa,πa] that make the integration with respect to *y* feasible in Equation (46). For small values of the parameter *q*, which is exactly our case, this Mathieu function has a simple form of the positive periodic function [55]

(45) $${\mathrm{ce}}_{0}\left(z,q\right)=2^{-1/2}\left[1-\left(q/2\right)\cos\left(2z\right)+O\left(q^{2}\right)\right].$$

Eventually, we arrive at the stationary state, which is the non-equilibrium stationary state (NESS) [58] of the relaxation process in the side-branched part of the comb system. Taking into account that χ(y) is a symmetrical function, we obtain it as follows:

(46) $$g\left(y,s\right)=g\left(y\right)=e^{-\sqrt{s/d}\left|y\right|}\chi \left(y\right)=u\left(y\right)=\frac{1}{\sqrt{2}}\left[1-\frac{3V_{0}}{2d}\cos\left(2\pi y\right)\right].$$

This also yields $g\left(0\right)∼\left(1-3V_{0}/\left[2d\right]\right)/\sqrt{2}$.

### 3.3. PDF of the Comb Model

Collecting the results of this section for the comb PDF P(x,y,t) given in Equations (28) and (29), we first present its Laplace-image form as follows:

(47) $$\hat{P}\left(x,y,s\right)=e^{-V(y)/[2d]}e^{-\left|y\right|\sqrt{s/d}}f\left(x,s\right)\chi \left(y,s\right)={\hat{P}}^{\left(0\right)}\left(x,y,s\right)\cdot e^{-V(y)/[2d]}\chi \left(y,s\right)$$

where ${\hat{P}}^{\left(0\right)}\left(x,y,s\right)=e^{-\left|y\right|\sqrt{s/d}}f\left(x,s\right)$ is the Laplace image of the standard comb model, considered in Section 2. This result can be easily extended to the half-plane comb model, considered in Section 2.1, by taking into account that the half-plane comb PDF ${\hat{P}}^{\left(0\right)}\left(x,y,t\right)$ is according to Equation (13). Note also that in this case, the number of transporting particles is not conserved due to the constant source term at the boundary with x=0.

The contribution of the side-branched periodic potential V(y) to the backbone transport reduces to the additional multiplication by the NESS, which is the side-branched exponential and the Mathieu function, $e^{-V(y)/[2d]}\;{\mathrm{ce}}_{0}\left(y\right)$.

**Figure 2:**
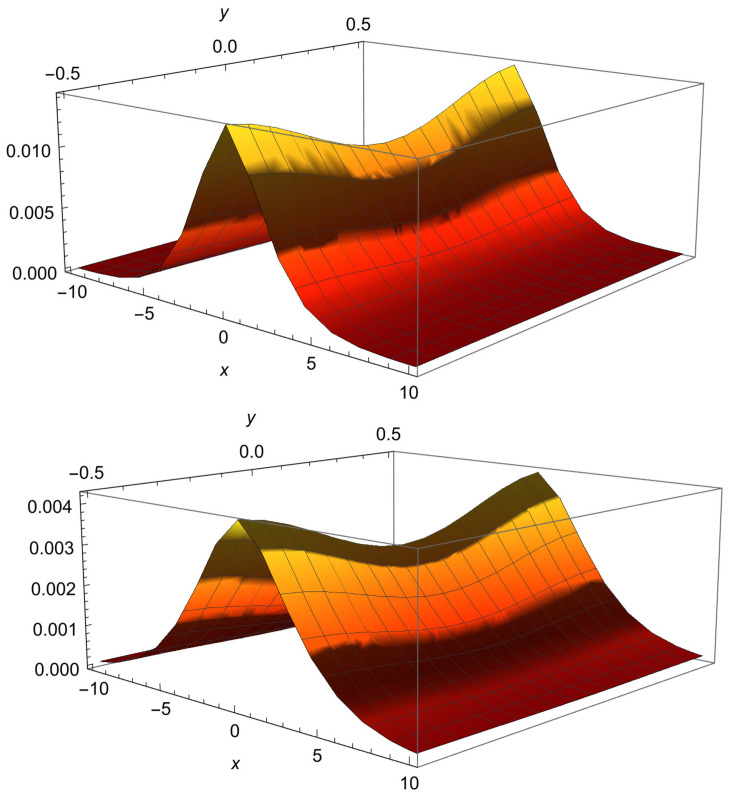
PDF (48) for $V_{0}=0.1$, d=1, a=1; top: t=10; bottom: t=50.

The asymptotic comb PDF is

(48) $$P\left(x,y,t\right)=L^{-1}\left[\hat{P}\left(x,y,s\right)\right]\propto e^{-V(y)/[2d]}\;{\mathrm{ce}}_{0}\left(y\right)\;L^{-1}\left[\frac{e^{-{|x|\left(4sd\right)}^{1/4}}}{2\sqrt{\sqrt{4sd}}}\right],$$

which consists of subdiffusion along the backbone and asymptotic (t→∞) NESS along the fingers. In this case, to normalize the PDF, the boundary conditions for the fingers should be reformulated and taken to be free boundary conditions at |y|=a/2. Eventually, it results in the direct product of the periodic in the *y* stationary distribution and the Fox *H*-function, as considered in Appendices Appendix A and Appendix B. Consequently, the Fox *H*-function here results from the inverse Laplace transform and is given by

(49) $$f\left(x,t\right)=L^{-1}\left[\frac{e^{-{|x|\left(4sd\right)}^{1/4}}}{2\sqrt{\sqrt{4sd}}}\right]=2{\left(4dt^{3}\right)}^{-1/4}H_{1,1}^{1,0}\left[\frac{4dx^{4}}{t}\left|\begin{array}{c} \left(1/4,1\right)\\ \left(0,4\right) \end{array}\right.\right]=t^{-3/4}\bar{f}\left(x^{4}/t\right);$$

see Equation (A3), where we use μ=ν=1/4 and $b={|x|\left(4d\right)}^{1/4}$. Graphical representation of the relaxation process at the large time asymptotics is shown in Figure 2, which pertains to Equations (48) and (49). Therefore, the scaling variable is $\eta =x/t^{1/4}$, and the MSD along the backbone is

(50) $$\langle x^{2}\left(t\right)\rangle =\frac{1}{N(t)}\int \left(x^{2}t^{-1/2}\right)\bar{f}\left(x^{4}t^{-1}\right)d\left(xt^{-1/4}\right)=\frac{1}{N(t)}\int \eta^{2}\bar{f}\left(\eta^{4}\right)d\eta =\frac{\mathrm{const}}{N(t)}.$$

This result leads to subdiffusion, since the total probability N(t) on the backbone is not conserved.

(51) $$N\left(t\right)=t^{-1/2}\int \bar{f}\left(x^{4}t^{-1}\right)d\left(xt^{-1/4}\right)=\mathrm{const}\times t^{-1/2}.$$

Therefore, the MSD reads $\langle x^{2}\left(t\right)\rangle =\mathrm{const}\times t^{1/2}$.

## 4. Summary

In this paper, a comb model with a periodic potential in fingers has been considered. Our main findings can be summarized as follows. Firstly, we have shown that the periodic motion in fingers affects the anomalous diffusion along the backbone, leading, under appropriate boundary conditions, to the occurrence of a non-equilibrium stationary state (NESS). Secondly, we have seen that the distribution of the probability density in the vicinity of NESS is given by a variant of the Mathieu function. Thirdly, we have inferred the asymptotic transport scalings for the backbone transport (Section 2.1) and showed that the transport is subdiffusive, with the mean-squared particle displacement growing with time as $\propto t^{1/2}$. These basic findings need to be augmented with some explanatory notes as follows.

Our first note here is concerned with the side-branched process described by Equation (41), with the solution $\chi \left(y,s\right)=e^{\sqrt{s/d}\left|y\right|}\;{\mathrm{ce}}_{0}\left(y\right)$. By its construction, Equation (41) describes the particle motion within the interval $y\in \left[-1/2,0^{-}\right)\cup \left(0^{+},1/2\right]$. The point y=0 is excluded from the consideration, since the corresponding term belongs to the backbone dynamics and is taken into account in Equation (35) for f(x,s). However, in the final PDF (48), the Mathieu function ${\mathrm{ce}}_{0}\left(y\right)$ is already defined at y=0, as well, since the final expression (48) is valid for all values of *y*, which is also taken into account by g(0).

The infinite strip $\left(x,y\right)\in R_{1}\times 1$, where the periodic potential affects the comb diffusion, is leaked at the boundaries y=±1/2. This results from the periodicity of the stationary distribution $g\left(y\right)=e^{-V(y)/[2d]}\;{\mathrm{ce}}_{0}\left(y\right)$, when $g\left(1/2+2\pi n\right)=g\left(1/2\right)=e^{V_{0}/\left[2d\right]}\left[1+4V_{0}/d\right]/\sqrt{2}$. Therefore, there are free boundary conditions for the fingers. It should be pointed out that this stationary solution is asymptotic and valid for large times, since initially it is a superposition of a complete set of the Mathieu functions, which form the initial δ(y) function due to the completeness property of the periodic Mathieu functions [59]. However, as follows from Equation (41), the spectrum α∝−s is negative; that is, it is bound, and tends to zero for the large-time (s→0) asymptotics. As a result, this periodic asymptotic solution is described by the Mathieu function ${\mathrm{ce}}_{0}\left(y,q\to 0\right)$.

Last but not least, it is suggested that the proposed comb model mimics the defining features of anisotropic transport in beta–plane atmospheric (drift–wave plasma) turbulence. Support for this suggestion can be found in the analysis of Refs. [45,46], where one associates the *y* direction with the direction along the flow, and the *x* direction with the direction perpendicular to the flow. In this context, the comb model provides a simplified yet relevant theoretical framework to characterize the inherent coupling between turbulent degrees of freedom, with the periodic potential along the *y* direction affecting the anomalous transport along the *x* direction. The finding above that the MSD along *x* grows with time as $t^{1/2}$ can be supported by the results of computer simulations of drift–wave anisotropic plasma turbulence, reported in [60,61].

Developing these viewpoints, one might further associate the NESS solutions of the comb model with turbulence-dominated, long-lived layered structures, zonal flows and staircases, see Ref. [47] and references therein. In this context, the periodic potential in fingers has a very clear physical interpretation: it captures the extra harmonics (in the poloidal cross-section) resulting from bending into a torus of otherwise cylindrically symmetric magnetic flux surfaces, the so-called shape effect (and thus the effect of toroidicity and flux-surface shaping on the poloidal plasma convection in a tokamak, e.g., Ref. [62]). Also, in the optics of NESS, it emphasizes the crucial role of toroidal geometry in the occurrence of layered structures and staircases; see Refs. [41,42,45].

Overall, the comb model opens up a new perspective on the study of staircase self-organization using fundamental methods. Extending this study to fluid (and fluid-like, such as electrostatic drift–wave) applications might be strongly advocated. In this connection, we would like to remark that the comb model correctly describes the transport of drift waves in a reactor-relevant setup of fusion plasma [46].

## Data Availability

No new data were created because this is a theoretical article and the authors choose has statement.

## Abbreviations

The following abbreviations are used in this manuscript:

PDF	Probability density function
DF	Density function
MSD	Mean squared displacement
w.r.t.	with respect to

## Appendix A. Laplace-Mellin Transformation: Mellin-Barnes Integral

Let us estimate the inverse Laplace transform of a general expression like $\hat{R}\left(s\right)=s^{-\mu }e^{-bs^{\nu }}$, where b>0, 0<ν<1 and μ is real. To execute this procedure, we follow Ref. [63] and perform first the Mellin transform of $\hat{R}\left(s\right)$ and then present it in the form of the inverse Mellin transform. Then we have for s>0

(A1) $$\begin{array}{cc} R^{*}\left(\xi \right) & =M\left[\hat{R}\left(s\right)\right]\left(\xi \right)=\int_{0}^{\infty }s^{\xi -1}\hat{R}\left(s\right)ds\\ & =\int_{0}^{\infty }s^{-\mu }e^{-bs^{\nu }}s^{\xi -1}ds=\frac{b^{(\mu -\xi )/\nu }}{\nu }\int_{0}^{\infty }x^{\frac{-\mu +\xi }{\nu }-1}e^{-x}dx\\ & =\frac{b^{(\mu -\xi )/\nu }}{\nu }\Gamma \left(\frac{-\mu +\xi }{\nu }\right), \end{array}$$

where the variable change $x=bs^{\nu }$ is used and $\Gamma \left(\frac{-\mu +\xi }{\nu }\right)$ is a gamma function [55,57].

Therefore, the term $\hat{R}\left(s\right)$ in the form of the inverse Mellin transform reads
  (A2) $$\hat{R}\left(s\right)=\frac{1}{2\pi i}\int_{c-i\infty }^{c+i\infty }R^{*}\left(\xi \right)s^{-\xi }d\xi$$

Eventually, the inverse Laplace transform of R(s) reads

(A3) $$\begin{array}{cc} R(t) & =\frac{1}{2\pi i}\int_{c-i\infty }^{c+i\infty }e^{st}\hat{R}\left(s\right)=\frac{b^{\mu /\nu }}{\nu }\frac{1}{2\pi i}\int_{c-i\infty }^{c+i\infty }b^{-\xi /\nu }\Gamma \left(\frac{-\mu +\xi }{\nu }\right)\cdot \frac{1}{2\pi i}\int_{c-i\infty }^{c+i\infty }s^{-\xi }e^{st}ds\\ \; & =\frac{b^{\mu /\nu }}{\nu }\cdot \frac{t^{-1}}{2\pi i}\int_{c-i\infty }^{c+i\infty }\frac{\Gamma \left(-\mu /\nu +\xi /\nu \right)}{\Gamma (\xi )}{\left[b^{1/\nu }/t\right]}^{-\xi }d\xi \\ & =\frac{1}{\nu t^{1-\mu }}\frac{1}{2\pi i}\int_{c-i\infty }^{c+i\infty }\frac{\Gamma \left(\xi /\nu \right)}{\Gamma (\xi +\mu )}{\left[b^{1/\nu }/t\right]}^{-\xi }d\xi , \end{array}$$

where in the last line the variable change ξ−μ→ξ is used.

## Appendix B. Fox H-Function

The Fox *H*-function (or *H*-function) is defined by means of the following Mellin-Barnes integral [51,63]

(A4) $$H_{p,q}^{m,n}\left(z\right)=H_{p,q}^{m,n}\left[z\left|\begin{array}{c} \left(a_{1},A_{1}\right),...,\left(a_{p},A_{p}\right)\\ \left(b_{1},B_{1}\right),...,\left(b_{q},B_{q}\right) \end{array}\right.\right]=H_{p,q}^{m,n}\left[z\left|\begin{array}{c} \left(a_{p},A_{p}\right)\\ \left(b_{q},B_{q}\right) \end{array}\right.\right]=\frac{1}{2\pi ı}\int_{\Omega }\theta \left(s\right)z^{-s}\;ds,$$

where

(A5) $$\begin{array}{c} \theta \left(s\right)=\frac{\prod_{j=1}^{m}\Gamma \left(b_{j}+B_{j}s\right)\prod_{j=1}^{n}\Gamma \left(1-a_{j}-A_{j}s\right)}{\prod_{j=m+1}^{q}\Gamma \left(1-b_{j}-B_{j}s\right)\prod_{j=n+1}^{p}\Gamma \left(a_{j}+A_{j}s\right)}, \end{array}$$

0≤n≤p, 1≤m≤q, $a_{i},b_{j}\in C$, $A_{i},B_{j}\in R^{+}$, i=1,...,p, j=1,...,q. Contour integration Ω starts at c−ı∞ and finishes at c+ı∞, separating the poles of the function $\Gamma (b_{j}+B_{j}s)$, j=1,...,m, with those of the function $\Gamma (1-a_{i}-A_{i}s)$, i=1,...,n. It plays an important role in the theory of fractional differential equations enabling a closed-form representation of the solutions of fractional diffusion-wave equations. It is a very general function, which reduces o many special cases in the form of well-known special functions [17,63].

## References

[1] White S.R., Barma M. (1984). Field-induced drift and trapping in percolation networks. J. Phys. A Math. Gen..

[2] Gefen Y., Goldhirsch I. (1985). Biased diffusion on random networks: Mean first passage time and DC conductivity. J. Phys. A Math. Gen..

[3] Weiss G.H., Havlin S. (1986). Some properties of a random walk on a comb structure. Phys. A.

[4] Baldi G., Burioni R., Cassi D. (2004). Localized states on comb lattices. Phys. Rev. E.

[5] Baskin E., Iomin A. (2004). Superdiffusion on a Comb Structure. Phys. Rev. Lett..

[6] Dvoretskaya O.A., Kondratenko P.S. (2009). Anomalous transport regimes and asymptotic concentration distributions in the presence of advection and diffusion on a comb structure. Phys. Rev. E.

[7] Iomin A. (2011). Subdiffusion on a fractal comb. Phys. Rev. E.

[8] Forte G., Burioni R., Cecconi F., Vulpiani A. (2013). Anomalous diffusion and response in branched systems: A simple analysis. J. Phys. Condens. Matter.

[9] Rebenshtok A., Barkai E. (2013). Occupation times on a comb with ramified teeth. Phys. Rev. E.

[10] Agliari E., Blumen A., Cassi D. (2014). Slow encounters of particle pairs in branched structures. Phys. Rev. E.

[11] Sandev T., Iomin A., Kantz H., Metzler R., Chechkin A. (2016). Comb model with slow and ultraslow diffusion. Math. Model. Nat. Phenom..

[12] Sandev T., Iomin A., Kantz H. (2015). Fractional diffusion on a fractal grid comb. Phys. Rev. E.

[13] Sandev T., Iomin A., Méndez V. (2016). Lévy processes on a generalized fractal comb. J. Phys. A Math. Theor..

[14] ben Avraham D., Havlin S. (2000). Diffusion and Reactions in Fractals and Disordered Systems.

[15] Sokolov I.M. (2012). Models of anomalous diffusion in crowded environments. Soft Matter.

[16] Iomin A., Mèndez V., Horsthemke W. (2018). Fractional Dynamics in Comb-like Structures.

[17] Sandev T., Iomin A. (2022). Special Functions of Fractional Calculus.

[18] Iyer C., Barma M., Singh H., Dhar D. (2025). Asymmetric Simple Exclusion Process on the Percolation Cluster: Waiting Time Distribution in Side Branches. Phys. Rev. Lett..

[19] Lenzi E.K., Rosseto M.P., Gryczak D.W., de Souza P.A., Lenzi M.K., Ribeiro H.V., Zola R.S. (2025). Diffusion in comb-structured surfaces coupled to bulk processes. Chaos Interdiscip. J. Nonlinear Sci..

[20] Lin L., Chen S., Bao C., Feng L., Zheng L., Zhu J., Zhang J. (2023). Analysis of the absorbing boundary conditions for anomalous diffusion in comb model with Cattaneo model in an unbounded region. Chaos Solitons Fractals.

[21] Zhu Y., Yuan Z., Peng J. (2025). First passage properties of d-dimensional finite combs with different growth modes. Chaos Interdiscip. J. Nonlinear Sci..

[22] Traytak S.D. (2025). Fractional differentiation method: Application to the trapping reactions in the comb-like structures with relaxation. J. Chem. Phys..

[23] Venturelli D., Illien P., Grabsch A., Bénichou O. (2025). Dynamics of soft interacting particles on a comb. J. Phys. A Math. Theor..

[24] Iomin A. (2012). Superdiffusive comb: Application to experimental observation of anomalous diffusion in one dimension. Phys. Rev. E.

[25] Liu L., Chen S., Feng L., Wang J., Zhang S., Chen Y., Si X., Zheng L. (2023). Analysis of the anomalous diffusion in comb structure with absorbing boundary conditions. J. Comput. Phys..

[26] Sandev T., Iomin A. (2018). Finite-velocity diffusion on a comb. Europhys. Lett..

[27] Tateishi A., Ribeiro H., Sandev T., Petreska I., Lenzi E. (2020). Quenched and annealed disorder mechanisms in comb models with fractional operators. Phys. Rev. E.

[28] Iomin A. (2024). Non-Markovian quantum mechanics on comb. Chaos Interdiscip. J. Nonlinear Sci..

[29] Iomin A. (2006). Toy model of fractional transport of cancer cells due to self-entrapping. Phys. Rev. E.

[30] Iomin A. (2012). A toy model of fractal glioma development under RF electric field treatment. Eur. Phys. J. E.

[31] Santamaria F., Wils S., De Schutter E., Augustine G.J. (2006). Anomalous diffusion in Purkinje cell dendrites caused by spines. Neuron.

[32] Méndez V., Iomin A. (2013). Comb-like models for transport along spiny dendrites. Chaos Solitons Fractals.

[33] Iomin A. (2019). Richardson diffusion in neurons. Phys. Rev. E.

[34] Masó-Puigdellosas A., Sandev T., Méndez V. (2023). Random Walks on Comb-like Structures under Stochastic Resetting. Entropy.

[35] Trajanovski P., Jolakoski P., Kocarev L., Sandev T. (2023). Ornstein–Uhlenbeck Process on Three-Dimensional Comb under Stochastic Resetting. Mathematics.

[36] Trajanovski P., Jolakoski P., Zelenkovski K., Iomin A., Kocarev L., Sandev T. (2023). Ornstein-Uhlenbeck process and generalizations: Particle dynamics under comb constraints and stochastic resetting. Phys. Rev. E.

[37] Plyukhin A.V., Plyukhin D. (2017). Random walks on uniform and non-uniform combs and brushes. J. Stat. Mech. Theory Exp..

[38] Sandev T., Iomin A., Kocarev L. (2020). Hitting times in turbulent diffusion due to multiplicative noise. Phys. Rev. E.

[39] Dif-Pradalier G., Diamond P.H., Grandgirard V., Sarazin Y., Abiteboul J., Garbet X., Ghendrih P., Strugarek A., Ku S., Chang C.S. (2010). On the validity of the local diffusive paradigm in turbulent plasma transport. Phys. Rev. E.

[40] Dif-Pradalier G., Hornung G., Ghendrih P., Sarazin Y., Clairet F., Vermare L., Diamond P., Abiteboul J., Cartier-Michaud T., Ehrlacher C. (2015). Finding the elusive **E**× **B** staircase in magnetized plasmas. Phys. Rev. Lett..

[41] Dif-Pradalier G., Hornung G., Garbet X., Ghendrih P., Grandgirard V., Latu G., Sarazin Y. (2017). The **E** × **B** staircase of magnetised plasmas. Nucl. Fusion.

[42] Hornung G., Dif-Pradalier G., Clairet F., Sarazin Y., Sabot R., Hennequin P., Verdoolaege G. (2017). **E** × **B** staircases and barrier permeability in magnetised plasmas. Nucl. Fusion.

[43] Milovanov A.V., Rasmussen J.J. (2018). Lévy flights on a comb and the plasma staircase. Phys. Rev. E.

[44] Garbet X., Panico O., Varennes R., Gillot C., Dif-Pradalier G., Sarazin Y., Grandgirard V., Ghendrih P., Vermare L. (2021). Wave trapping and **E**× **B** staircases. Phys. Plasmas.

[45] Milovanov A.V., Rasmussen J.J., Dif-Pradalier G. (2021). Self-consistent model of the plasma staircase and nonlinear Schrödinger equation with subquadratic power nonlinearity. Phys. Rev. E.

[46] Milovanov A.V., Iomin A., Rasmussen J.J. (2025). Turbulence spreading and anomalous diffusion on combs. Phys. Rev. E.

[47] Hahm T., Diamond P. (2018). Mesoscopic transport events and the breakdown of Fick’s law for turbulent fluxes. J. Korean Phys. Soc..

[48] Arkhincheev V.E., Baskin E.M. (1991). Anomalous diffusion and drift in the comb model of percolation clusters. Sov. Phys. JETP.

[49] Iomin A., Zaburdaev V., Pfohl T. (2016). Reaction front propagation of actin polymerization in a comb-reaction system. Chaos Solitons Fractals.

[50] Crank J. (1975). The Mathematics of Diffusion.

[51] Fox C. (1961). The *G* and *H* functions as symmetrical Fourier kernels. Trans. Am. Math. Soc..

[52] Gorenflo R., Kilbas A.A., Mainardi F. (2020). Mittag-Leffler Functions, Related Topics and Applications.

[53] Risken H. (1996). The Fokker-Planck Equation.

[54] Defaveri L., Barkai E., Kessler D.A. (2023). Brownian particles in periodic potentials: Coarse-graining versus fine structure. Phys. Rev. E.

[55] Abramovitz M., Stegun I.A. (1972). Handbook of Mathematical Functions with Formulas, Graphs, and Mathematical Tables.

[56] Wolf G. (2025). Mathieu Functions and Hill’s Equation. NIST Digital Library of Mathematical Functions.

[57] Jahnke E., Emde F., Lösch F. (1960). Tables of Higher Functions.

[58] Gallavotti G., Cohen E.G.D. (2004). Nonequilibrium stationary states and entropy. Phys. Rev. E.

[59] Bateman H., Erdélyi A. (1955). Higher Transcendental Functions, Version 3.

[60] Basu R., Jessen T., Naulin V., Rasmussen J.J. (2003). Turbulent flux and the diffusion of passive tracers in electrostatic turbulence. Phys. Plasmas.

[61] Basu R., Naulin V., Rasmussen J.J. (2003). Particle diffusion in anisotropic turbulence. Commun. Nonlinear Sci. Numer. Simul..

[62] Beeke O., Barnes M., Romanelli M., Nakata M., Yoshida M. (2021). Impact of shaping on microstability in high-performance tokamak plasmas. Nucl. Fusion.

[63] Mathai A.M., Saxena R.K., Haubold H.J. (2010). The H-Function: Theory and Applications.

